# P-16. Comparison of Switching to Daptomycin versus Remaining on Vancomycin for Bacteremia due to Methicillin-Resistant Staphylococcus aureus in Patients who Inject Drugs

**DOI:** 10.1093/ofid/ofaf695.247

**Published:** 2026-01-11

**Authors:** Amanda Binkley, Sharon Tsay, Katherine Mersinger, Diana Walczyk

**Affiliations:** Penn Presbyterian Medical Center, North Wales, PA; University of Pennsylvania, Philadelphia, Pennsylvania; Hospital of the University of Pennsylvania, Philadelphia, Pennsylvania; MedStar Franklin Square Medical Center, Baltimore, MD

## Abstract

**Background:**

Injection drug use is an established risk factor for invasive infections caused by methicillin-resistant *Staphylococcus aureus* (MRSA). People who inject drugs (PWID) who are admitted to the hospital for severe infections are typically initiated on empiric intravenous (IV) vancomycin for MRSA coverage. However, it can be difficult to perform vancomycin therapeutic drug monitoring and achieve pharmacokinetic targets in this patient population. Thus, in some cases, patients are anecdotally transitioned to alternative antibiotics that can be administered less frequently and are not subject to therapeutic drug monitoring, such as IV daptomycin.

**Figure:**
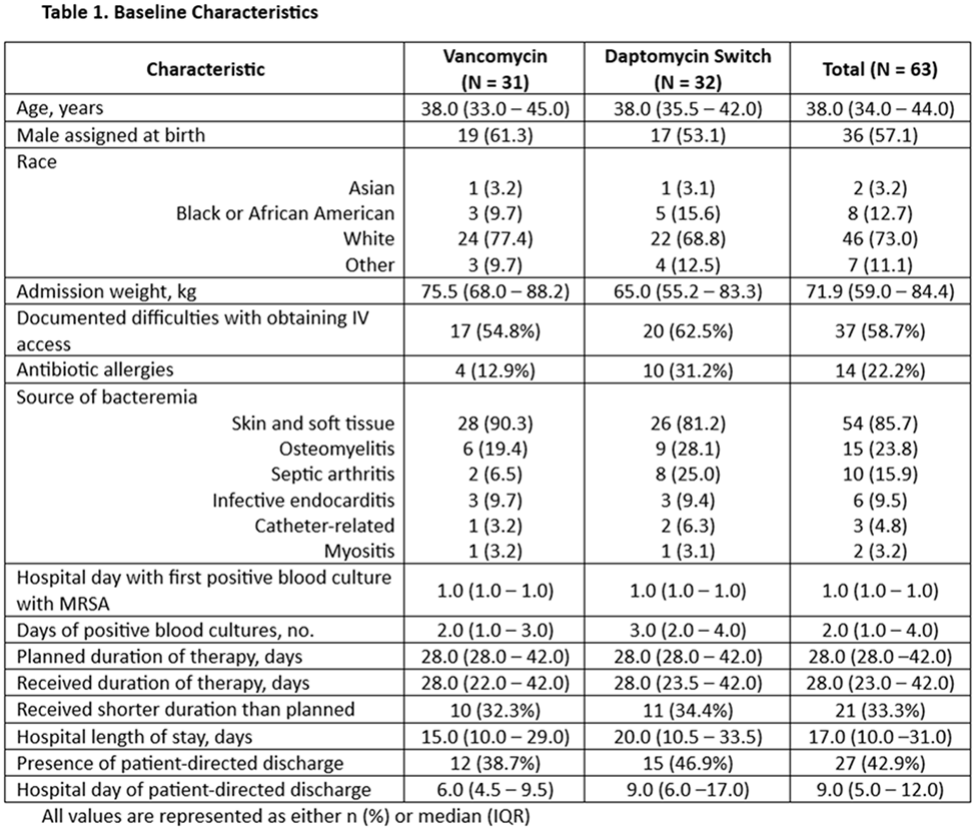

**Figure:**
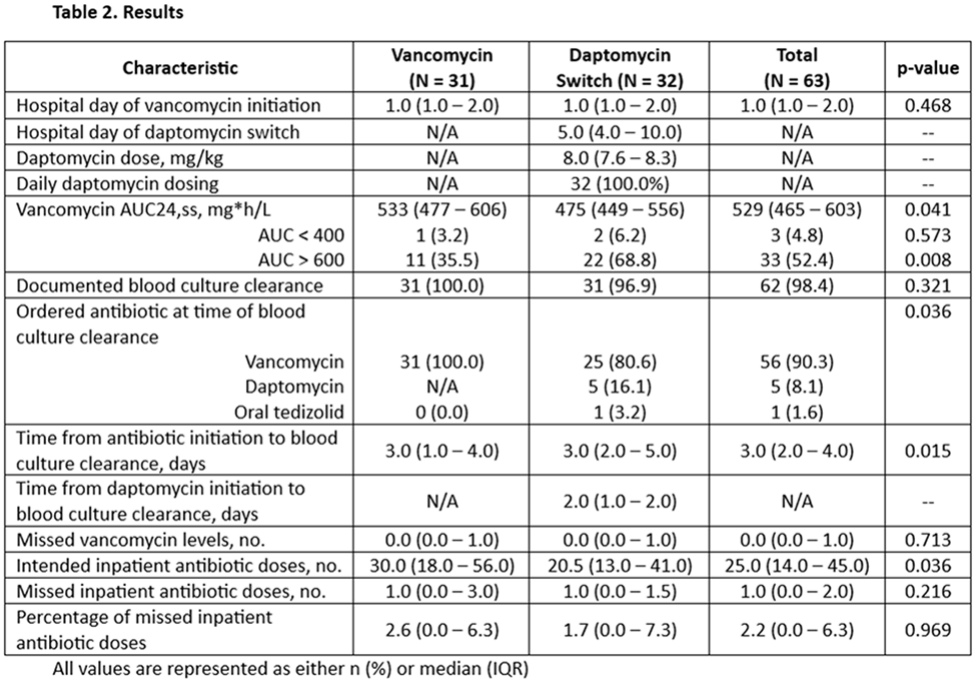

**Methods:**

We performed a retrospective, dual-center study evaluating antibiotic practices in adult PWID admitted with MRSA bacteremia between September 1, 2019 and September 1, 2024 who received IV vancomycin for at least 48 hours. The primary objective was the incidence of switching from vancomycin to daptomycin. Secondary objectives included the timing of antibiotic modification, time to blood culture clearance, and other factors associated with the switch.

**Figure:**
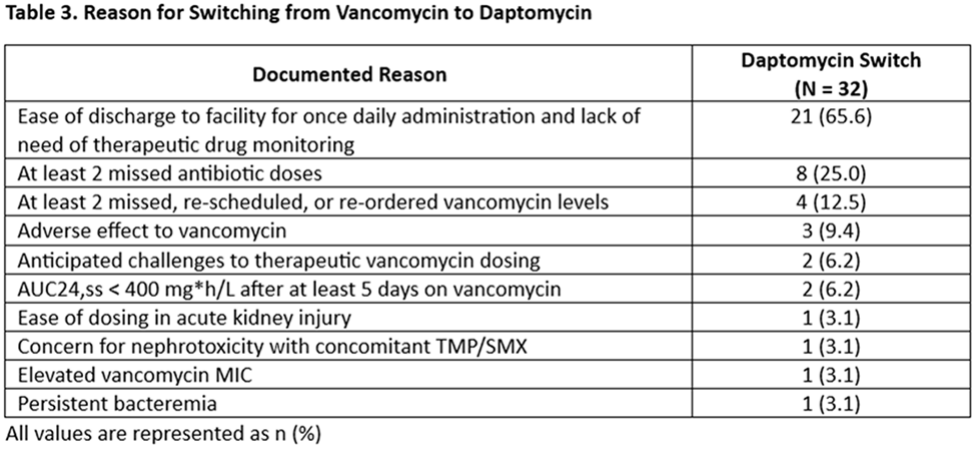

**Results:**

A total of 63 patients met eligibility criteria and were included in the analysis. The transition from vancomycin to daptomycin occurred in 32 (50.8%) patients after a median of 5.0 days (IQR 4.0 – 10.0) on empiric vancomycin. The median daptomycin dose administered was 8.0 mg/kg (IQR 7.6 – 8.3) daily. Overall time from initiation of antibiotics with MRSA activity to blood culture clearance was 3.0 days (IQR 2.0 – 4.0). Blood culture clearance occurred after a median of 2.0 days on daptomycin (IQR 1.0 to 2.0). Of the patients transitioned to daptomycin, the most common reasons for switch were ease of discharge to a facility for once daily administration and lack of need for therapeutic drug monitoring.

**Conclusion:**

Approximately half of the included patients were transitioned from vancomycin to daptomycin for treatment of MRSA bacteremia. This is consistent with concerns in clinical practice relating to safety, efficacy, and monitoring of vancomycin in this patient population. Given how commonly this occurs, these findings suggest it may be beneficial for institutions to develop guidelines for the treatment of MRSA bacteremia in PWID.

**Disclosures:**

Amanda Binkley, PharmD, BCIDP, AAHIVP, Shionogi: Advisor/Consultant Katherine Mersinger, PharmD, Melinta Therapeutics: Honoraria

